# Lipoprotein hydrophobic core lipids are partially extruded to surface in smaller HDL: “Herniated” HDL, a common feature in diabetes

**DOI:** 10.1038/srep19249

**Published:** 2016-01-18

**Authors:** Núria Amigó, Roger Mallol, Mercedes Heras, Sergio Martínez-Hervás, Francisco Blanco-Vaca, Joan Carles Escolà-Gil, Núria Plana, Óscar Yanes, Lluís Masana, Xavier Correig

**Affiliations:** 1 Metabolomics Platform, Department of Electronic Engineering, Rovira i Virgili University, IISPV, Av. PaÏsos Catalans 26, 43007, Tarragona, Spain; 2 Spanish Biomedical Research Centre in Diabetes and Associated Metabolic Disorders (CIBERDEM), C. Monforte de Lemos 3-5, 28029, Madrid, Spain; 3 Vascular Medicine and Metabolism Unit, Research Unit on Lipids and Atherosclerosis, Sant Joan University Hospital, Rovira i Virgili University, IISPV, C. Sant Joan s/n, 43201, Reus, Spain; 4 Endocrinology and Nutrition Department, Hospital Clinico Universitario, INCLIVA, Department of Medicine, University of Valencia, Av. Blasco Ibañez 17, 46010, Valencia, Spain; 5 Institut d’Investigacions Biomèdiques (IIB) Sant Pau, Antoni M. Claret 167, 08025, Barcelona, spain; 6 Departament de Bioquímica i Biologia Molecular, Universitat Autònoma de Barcelona (UAB), Edifici M. Campus de la UAB, 08193, Bellaterra, Spain

## Abstract

Recent studies have shown that pharmacological increases in HDL cholesterol concentrations do not necessarily translate into clinical benefits for patients, raising concerns about its predictive value for cardiovascular events. Here we hypothesize that the size-modulated lipid distribution within HDL particles is compromised in metabolic disorders that have abnormal HDL particle sizes, such as type 2 diabetes mellitus (DM2). By using NMR spectroscopy combined with a biochemical volumetric model we determined the size and spatial lipid distribution of HDL subclasses in a cohort of 26 controls and 29 DM2 patients before and after two drug treatments, one with niacin plus laropiprant and another with fenofibrate as an add-on to simvastatin. We further characterized the HDL surface properties using atomic force microscopy and fluorescent probes to show an abnormal lipid distribution within smaller HDL particles, a subclass particularly enriched in the DM2 patients. The reduction in the size, force cholesterol esters and triglycerides to emerge from the HDL core to the surface, making the outer surface of HDL more hydrophobic. Interestingly, pharmacological interventions had no effect on this undesired configuration, which may explain the lack of clinical benefits in DM2 subjects.

The International Diabetes Federation recently announced that, prior to 2014, over 387 million people had been diagnosed with diabetes^1^. Type 2 diabetes mellitus (DM2) accounts for at least 90% of the cases of diabetes and it is increasing every year due to genetic factors and changes in lifestyle. A typical feature of DM2 – as well as obesity, insulin resistance and the metabolic syndrome – is atherogenic dyslipidemia, which has emerged as an important risk factor for cardiovascular disease (CVD)^2^. Atherogenic dyslipidemia consists of a triad of increased blood concentrations of small and cholesterol-depleted low-density lipoprotein (LDL) particles, decreased high-density lipoprotein cholesterol (HDL-C) and increased total triglycerides. It should be noted that it has become established therapeutic practice to increase HDL-C concentrations to decrease cardiovascular risk. However, despite the strong inverse association of HDL-C plasma levels with coronary heart disease found in epidemiological studies^3^,^4^, recent evidence has raised serious concerns about the ability of HDL-C concentration to assess cardiovascular risk and, hence, whether it should be a good target for therapeutic interventions^5^,^6^. Indeed, several recent clinical trials involving therapeutic elevation of HDL-C were prematurely terminated on the basis of futility^7^,^8^,^9^.

Recent structure-function studies have suggested that HDL-C is not such a good marker of cardiovascular risk because HDL concentration does not always reflect HDL function^9^,^10^,^11^. In some circumstances the potentially protective functions of HDL may be compromised despite high concentrations of HDL-C^12^. This highlights the need to characterize lipoprotein particles using a range of additional parameters such as size, particle number and chemical composition^13^, which is expected to improve the assessment of CVD risk and guide lipid-lowering therapies^14^.

In this context, the structure of lipoproteins has been inferred to date from compositional analyses using the classical theoretical description of Shen and colleagues^15^, by which the structure of circulating lipoproteins is consistent with a spherical model of radius *r* in which a spherical liquid core of cholesterol esters and triglycerides is surrounded by a monolayer of free cholesterol and phospholipids, with proteins closely packed with the hydrophilic head groups of phospholipids on the outer surface of the particle. On the basis of an updated version of the spherical model proposed by Shen *et al*. and experimental data on HDL by ^1^H-NMR spectroscopy, the theoretical size of HDL particles has been estimated and a positive correlation found with the HDL-C/ApoA-I ratio^16^. In this regard, the emergence of experimental techniques mainly based on NMR spectroscopy makes it possible to characterize the size and particle number of lipoprotein particles^16^,^17^. This opens up a new scenario for studying lipoprotein structure and function. Specific HDL sizes (i.e., subclasses) and, in particular, the balance between large and small HDL particles seems to be important for evaluating cardiovascular risk^13^. Recent studies suggest that HDL particle number (HDL-P) may be a more suitable and independent risk factor than HDL-C^18^,^19^ or a combination of both parameters, HDL-C/HDL-P, can determine the antiatherogenic function of HDLs, rather than either parameter alone^20^.

Yet despite these advances, if HDL particle size and number are to be used in clinical practice for cardiovascular risk management, the ability of existing techniques to estimate consistent HDL size, concentration and lipid content needs to be evaluated. Interestingly, the development of these novel techniques has revealed that there is a discrepancy between the absolute values of HDL-P obtained by NMR and the values generated using the classical model of spherical lipoprotein structures proposed by Shen^15^. In particular, determination of HDL-P by NMR gives higher particle numbers than simulations based on the Shen model^16^. This discrepancy between experimental and theoretical data has led to modifications being made to the classical Shen model^21^,^22^. However, none of these studies have investigated the clinical implications of changes in the HDL structure.

Our study aims to gain greater insight into how some of the aforementioned HDL parameters are related (for example, the mean HDL size and the HDL subclass distribution), and how these parameters are affected by the lipid and protein concentrations in the healthy and the DM2 pathological state with atherogenic dyslipidemia. For this purpose, we used classical biochemical enzymatic techniques and advanced ^1^H-NMR spectroscopy, atomic force microscopy (AFM) and fluorescence experiments to model the HDL structure and to characterize the size, and the molecular composition and distribution of the HDL fraction. We also determined the balance between the three main HDL subclasses – namely, large, medium and small HDL – and characterized the differences in surface hydrophobicity between them. Finally, we performed the same analysis on the DM2 group after two pharmacological interventions with fenofibrate and niacin, respectively.

## Results

### HDL fraction analysis: from the biochemical composition and size of HDL fractions to the modification of the Shen model

**Figure 1:**
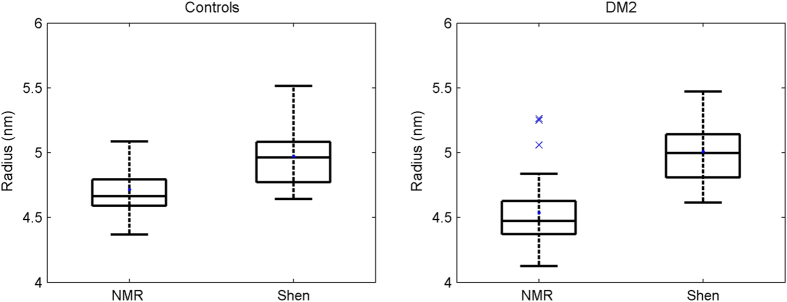
Differences between the mean HDL radius measured by NMR and estimated by the Shen model. The figure illustrates the differences between the mean radius obtained from NMR data and the radius derived from the Shen model for the CT group (n = 26) and the DM2 patients (n = 29).

The biochemical information of the HDL fractions determined by enzymatic assays is described in Table 1. As expected for DM2 patients, with the characteristic feature of low HDL cholesterol, all the HDL constituent molecules were significantly lower for the DM2 group than for the CT group, except triglycerides that were significantly higher. Figure 1 illustrates the differences between the mean radius of the HDL fractions obtained from DOSY ^1^H-NMR data (R_N_) and the mean radius derived from the Shen model (R_S_) for the CT group and the DM2 patients (see Section 1, SI). We observed that in both groups, R_S_ were higher than expected for the mean size of the HDL fractions.

Considering all the samples together, the mean radius measured by NMR was 4.6 [4.4–4.7] nm –expressed as median ± [25–75] – which is consistent with previously reported HDL mean sizes considering the relative abundances of large, medium and small HDL particles^19^,^23^,^24^. On the other hand, the derived mean radius from the Shen model (see Materials and Methods Eq. 2) was 5.0 [4.8–5.1] nm in concordance to a distribution of HDL particles centred on large HDL particles. The p value between the R_N_ and R_S_ distributions was p < 0.001.

To solve the discrepancies between the biochemical composition and the experimental sizes, we newly modified the Shen model, increasing the geometrical ratio between surface volume (V_Shell_) and core volume (V_Core_) to obtain a better agreement between the biochemical composition and the experimental size, on the basis that smaller particles have a higher V_Shell_/V_Core_ than larger particles. This modification led to some of the traditionally core lipids were located in the lipoprotein shell (see Materials and Methods). Table 2 reports the main parameters obtained when our modified Shen model was used to analyse the HDL fractions. In agreement with current knowledge about lipoprotein disorders in DM2 patients, we found that the mean radius for the CT group determined by NMR was higher than for the DM2 group (4.7 nm and 4.5 nm, respectively) (p = 0.002). Interestingly, our modified model detected the presence of hydrophobic core lipids in the lipoprotein shell. The percentage of hydrophobic core lipids, basically esterified cholesterol (EC) and triglycerides (TG), occupying the external shell was 12% for the CT group, significantly less than the 20% found for the DM2 group (p = 0.016). This redistribution of the core lipids meant that 3% and 6% of the volume of the external shell was occupied by hydrophobic lipids in the CT and the DM2 groups, respectively (p = 0.011). In addition, the lipidic core composition was quite different: the percentage of the inner core occupied by TG was 30% for the CT group, much less than the 43% for the DM2 group (p < 0.001).

The study design determined the plasma triglycerides levels of the DM2 subjects, and hence the HDL triglyceride levels, were significantly higher than those of the CT group. Independently, the mean size of the DM2 subjects was significantly reduced. Both conditions, high triglycerides and reduced size, were associated with a higher percentage of the core lipids in the surface, as these variables are correlated (see Section 2, SI, Figs S1 and S2). In order to explore whether the redistribution of the core lipids in the surface was due to high TG or more specific reason for DM2, we analysed separately the association of the TG, the size and the percentage of core lipids in the surface for each group (see Section 2, SI, Fig. S3). This analysis revealed a different behavior between the CT and the DM2 groups: while the levels of TG were highly associated with the percentage of the core lipids in the surface for the CT group (Pearson correlation coefficient r = 0.81) this association was lost for the DM2 group (r = 0.35). In the same direction, only the CT group presented a clear and inverse association between the size and the TG levels (see Section 2, SI, Fig. S3). Therefore, the CT subjects with high TG levels presented a smaller size and, therefore, an increased percentage of core lipids in the surface. Alternatively, the levels of TG in the DM2 group were not associated with the size; being possible to find subjects with low TG levels, and a reduced size, presenting a high percentage of hydrophobic core lipids in the surface. DM2, thus, was a clinical factor that emphasized the percentage of the core lipids in the surface independently of the TG levels.

The above results indicate differences in the composition and location of the hydrophobic lipids in the surface shell. The leakage of the hydrophobic lipids TG and EC to the surface may contribute to the change in polarity of the HDL surface and, in particular, the DM2 group should have a more hydrophobic HDL surface than the CT group. In order to verify the hypothesis that some of the core lipids are located in the surface shell, we carried out some fluorescence experiments to evaluate the surface polarity of lipoproteins. The fluorescent membrane probes are highly sensitive to the polarity of lipid membranes and lipid monolayers. We investigated the surface polarity of a subgroup of 8 CT subjects and 4 DM2 patients with three different membrane probes: Patman, Prodan and Laurdan. It has been widely reported that the fluorescence spectrum of these three probes in lipid membranes and in native lipoproteins and lipoprotein models is highly affected by the polarity of the microenvironment: a more hydrophobic microenvironment produces a blue shift of the emission maximum position^25^,^26^,^27^.

The fluorescence spectra of all three probes showed the same tendency: the wavelengths of the emission maxima (λ_em_) of the DM2 group were blue shifted, indicating that the surface microenvironment was more hydrophobic than in the CT group (see Section 3, SI Fig. S4). The Prodan probe exhibited the clearest and best defined spectra, which made it possible to quantify the different behaviour between groups. The emission wavelength was λ_em_ = 436.1 nm for the CT group and λ_em_ = 434.1 nm for the DM2 group, (p = 0.02). To evaluate the dispersion of the measurements and facilitate the visualization of the differences between the CT and the DM2 group, we performed a principal component analysis (PCA) of the fluorescence raw data of two repetitions per sample (see Section 3, SI Fig. S5).

### Application of the modified Shen model to the study of the HDL subclasses

**Figure 2:**
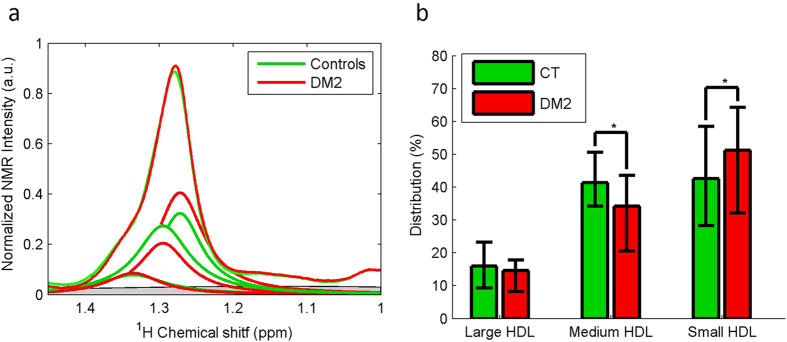
Balance between large, medium and small HDL subclasses of CT population and DM2 patients. (**a**) The figure illustrates the average of the normalised NMR spectra and the means of the three Lorentzian functions for the CT group (n = 26) and the DM2 patients (n = 29). The area under each curve represents the relative concentration of a particular subclass. (**b**) % of integrated area. Data are expressed as medians ± 25–75.

To further study the HDL fraction, we extended our modified Shen model to the three major HDL subclasses. We used the methylene signal of 1D diffusion-edited NMR spectra (LED) to obtain a qualitative picture of the distribution of particles in the three HDL subclasses (large, medium and small) that contain methylene groups in the constituting HDL molecules. The frequency at which the methylene groups of the lipids in lipoprotein particles resonate depends on the size of the particles that carry them. Larger particles resonate at higher frequencies^28^. We deconvoluted the methylene signal with a Lorentzian curve for each of the three major HDL subclasses (see Materials and Methods) to mathematically reproduce the raw NMR data and compute the peak area of the methylene signal. Because the NMR peak areas are proportional to the concentration of molecules in solution, the area below each Lorentzian curve of the methylene peak is proportional to the concentration of the methylene groups present in the cholesterol and triglyceride molecules carried by each HDL subclass and, hence, proportional to the HDL particles of each subclass. Figure 2 shows that the CT group presented a higher percentage of the medium HDL particle subclass than the DM2 group (41% and 34%, respectively) (p = 0.014). In contrast, the CT group presented a lower percentage of the small HDL subclass (42%) than the DM2 group (51%) (p = 0.041). The previous analysis of the mean sizes of the HDL fraction supported this distribution.

Since no data were available on the biochemical composition and size for each subclass we used the sizes and the concentrations of the molecules constituting each HDL subclasses described in the literature for healthy subjects^23^,^29^,^30^,^31^,^32^. We associated the large HDL subclass to HDL 2b, the medium HDL subclass to HDL 2a and the small HDL subclass to HDL 3^33^,^34^. The reported particle sizes were 6 nm, 5 nm and 4 nm for large, medium and small HDL respectively, and the molecular composition of each subclass is detailed in Table S1 (see Section 4, SI).

**Figure 3:**
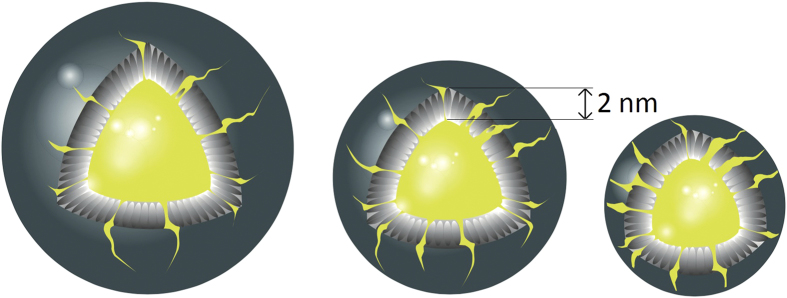
Structural model for different subclasses of HDL particles. The figure shows the differences in the amounts of core lipids located in the 2 nm shell depending on the lipoprotein size.

Once the biochemical composition and size had been assigned, our modified model was applied to each subclass. As can be seen in Fig. 3, our findings revealed that the small HDL subclass is much more affected by the space restrictions than the large or medium HDL subclasses. This has an effect on the proportion of core lipids in their surface shell. The resulting percentage of hydrophobic lipids increases as the size decreases: from 3% in the case of large HDL particles and 15% for the medium-sized particles, up to 56% for the smallest particles.

The mathematical models indicated that there were different amounts of hydrophobic lipids in the different HDL subclasses. We used Atomic Force Microscopy (AFM) to prove whether the surface polarity of lipoproteins depended on size. The size and the hydrophobicity of the HDL surface was qualitatively characterised as the adhesion force between the tip of the AFM probe (basically hydrophilic) and the lipoprotein surface depends on the hydrophobic or hydrophilic nature of the sensed surface^35^,^36^.

Just as the model did, the AFM experiments showed that smaller particles had a more hydrophobic surface. We analyzed three different samples, and they all presented the same tendency: the adhesion force between the hydrophilic tip and the HDL surface was higher for large particles, indicating a more hydrophilic environment. The relation between the size and the adhesion force is represented in Figure S6 (see Section 5, SI). For the three samples, the correlation coefficients were 0.34, 0.52 and 0.36 respectively (p values 0.23, 0.01 and 0.19), revealing that as larger particles were more hydrophilic surface presented (see Section 5, SI, Fig. 7). These qualitative results reinforce the hypothesis that the hydrophobic lipids in small particles rise to the surface.

### Characterization of the HDL fraction of DM2 subjects after fenofibrate and niacin treatments

Finally, we characterized the HDL fraction of the DM2 group using two different, commonly used treatments with fenofibrate and niacin. As in the case above, we first used our modified Shen model to measure the lipid and protein concentrations (see Table 3) and the mean HDL size and obtain the fraction of the core lipids located in the external shell. Independently, we determined the HDL distribution in the large, medium and small subclasses by NMR.

Table 4 shows that neither of the treatments had any significant effect on the mean size and percentage (and the composition) of core lipids in the external shell, or the subclass distribution of the DM2 group. However, the effects of the treatments were quite different: the fenofibrate treatment tended to decrease the mean HDL size and the niacin treatment tended to increase it, leading to a statistically significant change in size when both treatments were compared (p = 0.042).

**Figure 4:**
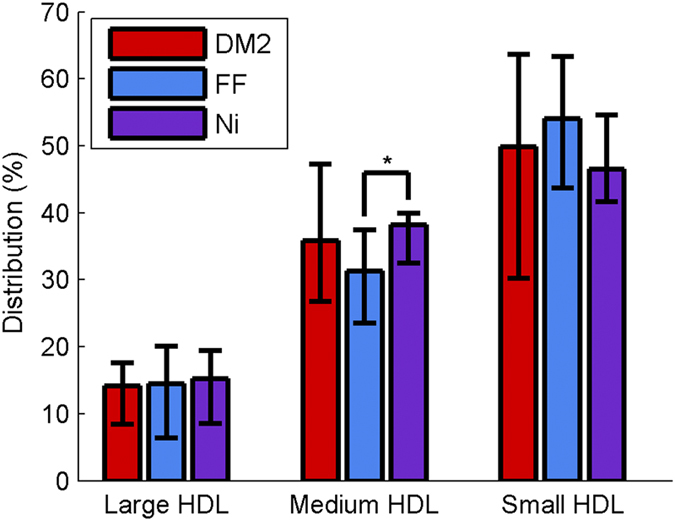
Treatment effects on the balance between large, medium and small HDL subclasses of DM2 patients (% integrated area). Data are expressed as medians ± 25–75 of the 24 DM2 subjects who finished both interventions. FF: fenofibrate, Ni: niacin.

As far as mean size is concerned, Fig. 4 shows that the fenofibrate treatment tended to increase the relative concentration of the small HDL subclass, and that the niacin treatment tended to increase the relative concentration of the medium HDL subclass. These different effects were statistically significant when both treatments were compared (p = 0.015).

## Discussion

The current need to characterize HDL particles using a range of additional parameters such us size, particle number, subclass distribution and chemical composition increases since HDL-C does not always reflect HDL antiatherogenic function. Lipidomic and structural-function approaches suggest that the alterations of the lipidome^37^ and the negatively charged enrichment of phospholipids^38^ in the smaller HDL subfraction play a crucial role for HDL dysfunctionality.

Alternatively, the preceding analysis strongly supports the hypothesis that HDL functionality may be compromised in a pathological state such as DM2. Our results indicate that the HDL of a CT group is clearly different from that of a DM2 group, essentially due to the differences in HDL particle size and biochemical composition, and the implications for lipid relocation.

A reduction in size has consequences for the lipid distribution of HDL particles: Lipoproteins are spheres with a shell thickness of 2 nm that define an inner core cavity with a fixed volume. Therefore molecules are subject to spatial restrictions. The smaller the particle is, the tighter those spatial restrictions are. Thus, providing a lesser volume for hydrophobic lipids to rely on. Since volume of a sphere is proportional to the third power of its radius, the core volume is extremely sensitive to small changes in the radii. Consequently, as the pathological group has a smaller size, this phenomenon is emphasized.

To solve these spatial inconsistencies, we assumed that some of the lipids that are traditionally found inside the core (esterified cholesterol and triglycerides) have to rise to the lipoprotein surface for volumetric reasons. Other modifications in the spherical model of lipoprotein structure have been reported previously: the spherical shape becomes more discoidal or cylindrical^39^, free cholesterol can be found in the inner core^21^,^22^, and proteins are closely packed on the outer surface of the particle^15^. All these three modifications increase the external shell volume and diminish the internal core volume. Consequently, the fraction of hydrophobic core lipids outside the lipoprotein shell also increases.

One of the limitations of our model is to consider that the entire volume of the apolipoproteins fills the surface shell, independently of the curvature radius. Energy derived computational models studying the stability of lipoprotein structure identify that the penetration of the alpha helixes into the lipoprotein shell is modulated by the curvature radius, since them fill the space between the phospholipids head-groups^40^,^41^. The penetration of the amphiphilic helix alpha into the lipoprotein shell get higher as the size of the particles get smaller and more apolipoproteins are required to fill the gaps between phospholipids head-groups. The size-modulated integration of the apolipoprotein into the shell in the model would even more underestimate the surface volume of the HDL particles increasing the fraction of the hydrophobic core lipids in the surface.

Another limitation of this study is to not consider the particular concentration and composition of the fatty acids (FA) chains constituting the TG and CE molecules. Also, hydrophobicity of TG is less than that of CE so that TG tends to be more readily exposed to the lipid-water interface. Furthermore, the TG exposure to the surface is likely to be dependent on its miscibility in the phospholipid monolayer^42^. For that reason, the concentration and, particularly, the composition of the FA chains, might have an effect on the whole surface polarity of the HDL particles. Therefore, the change on the hydrophobicity of the small HDL particles induced by the presence of the core lipids in the surface in the DM2 group may be slightly reduced due to the increased concentration of TG molecules in the HDL particles. However, the modifications of the polarity due to the composition of the core lipids can be considered as a second order effect because CE is the major component for both, the CT and the DM2 group.

Fluorescence studies supported our hypothesis by providing experimental evidence that confirms that DM2 HDL particles have a different surface polarity. In our opinion, fluorescence is the only technique able to distinguish the polarity of the immediate surroundings of phospholipids because fluorescence dyes can be found among phospholipid molecules. The changes in the probe microenvironment are caused by the presence of hydrophobic lipid molecules instead of possible protein posttranslational modifications associated with the pathological state.

Both the high concentration of triglycerides in the HDL of the DM2 group and the higher number of small particles – which present the most abnormal lipids distribution – may explain the dysfunctional behaviour of HDL communicated by several researchers^43^,^44^,^45^. Moreover, the lipid composition of the hydrophobic lipids that leakage to the surface is significantly different^44^. The high concentration of triglycerides found in the DM2 group could accentuate even more the changes in the surface polarity. The mechanisms behind these HDL changes are not well established. We communicated^44^ that this group of diabetic patients have higher cholesterol-ester transfer protein activity, what could contribute to HDL smaller size and triglyceride enrichment.

The hydrophobic and hydrophilic forces that regulate the molecular interaction between lipoproteins, enzymes and cell membranes, and the conformation of apolipoproteins may be compromised^45^,^46^,^47^.

Surprisingly, despite the benefits of the two treatments on the lipid content – lower triglyceride count and higher cholesterol –the pathological state is not totally reversed after 12 weeks treatment with either fenofibrate or niacin, although we cannot exclude a more extensive HDL reparation after longer intervention periods. One possible reason for this is that the changes in the HDL triglyceride concentration and the mean radius that modulates the lipoprotein particle distribution are not enough. Instead, they hamper the correct distribution of HDL lipids and force them to leakage to the external lipoprotein shell like a *lipidic hernia*.

## Methods

### Study subjects

A complete description of the population has recently been published^44^. Briefly, 29 type 2 diabetic patients (DM2) were recruited: 18 male and 11 female, aged between 30 and 70 years old, and with HDL not exceeding 50 mg/dl in men or 60 mg/dl in women. The exclusion criteria were as follows: smoker, diagnosed with diabetes less than three months before, triglyceride levels above 400 mg/dl, glycated hemoglobin higher than 9%, albuminuria above 300 mg/mg creatinine, chronic kidney disease (estimated glomerular filtration rate <30 ml/min/1.73 m2), advanced retinopathy, neuropathy, cardiovascular disease in the last three months, chronic liver insufficiency, neoplastic disease or any chronic or incapacitating disease. The CT group consisted of 26 age- and gender-matched subjects without diabetes and with HDL cholesterol higher than 40 mg/dl for men or 50 mg/dl for women. After a 6-week lipid-lowering drug wash-out, the patients with type 2 diabetes were randomly distributed into two groups. One group received 20 mg simvastatin plus 145 mg fenofibrate, and the other group received 20 mg simvastatin plus 2 g niacin plus laropiprant for a 12-week period. After a washout period of 6 weeks, they interchanged the treatments for 12 weeks more. At the end of the study, 24 DM2 patients finished both treatments after 5 discontinued interventions.

Fasting blood samples were collected in EDTA tubes and centrifuged immediately for 15 min at 4 °C at 1500 x g at the basal point, after each intervention period in the DM2 group, and at the basal point in the CT group. Plasma samples were then kept at −80 °C until further analysis. The entire study and all related experimental protocols were approved by the Ethical Committee of the Sant Joan University Hospital (Reus, Spain) and the Ethical Committee of the Clinical University Hospital (Valencia, Spain). All of the subjects provided their written informed consent before participating in the study. The study was carried out in accordance with the standards set by the Declaration of Helsinki and Good Clinical Practice guidelines.

### Lipoprotein fractionation and HDL analysis

Prior to the NMR analysis, total HDL was isolated from plasma using sequential preparative ultracentrifugation at 1.21 g/ml density using previously described techniques^48^. Ultracentrifuged HDL (ucHDL) fractions were stored at −80 °C until the biochemical studies were carried out. In the ucHDL fraction, cholesterol, triglycerides, total protein, phospholipids and apolipoproteins were quantified using enzymatic and nephelometric assays adapted to a COBAS 6000 autoanalyzer (Roche Diagnostics, Rotkreuz, Switzerland).

### NMR experiment

^1^H NMR spectra were recorded on a Bruker Avance III 600 spectrometer operating at 310 K using two different pulses: a 2-D double stimulated echo (DSTE) pulse program with 16 bipolar gradients to obtain the diffusion coefficients of HDL fraction as previously reported^49^; and a 1D diffusion-edited pulse sequence with bipolar gradients and the longitudinal eddy-current delay (LED) scheme with two spoil gradients to determine the particle distribution between HDL subclasses. In both cases, the relaxation delay was 2 seconds and the Free Induction Decays (FIDs) were collected into 64 Kcomplex data points and 64 scans per sample.

***Radius extraction:*** The DSTE methyl signal was fitted with one Lorentzian curve to obtain the averaged diffusion coefficient (*D*_*Coef*_) of the lipoprotein particles as previously reported^50^. Then, the hydrodynamic radii of the lipoprotein fractions (*R*_*H*_) were extracted from the Stokes-Einstein equation:


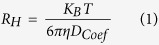


where *K*_*B*_ is the Boltzmann constant, *T* is the temperature (310 K), and *η* is the mean viscosity of the solution (0.75 ± 0.02 mPa·s) measured at 310 Kwith a Cannon-Manning semi-micro capillary viscometer, in agreement with previous work^49^.

### Particle size distribution

To obtain a qualitative picture of the size distribution of the HDL lipoproteins, we used the methylene signal from the LED NMR experiments. The methylene signal had enough resolution to be fitted with three Lorentzians, one for each of the three different HDL subclasses (large, medium and small HDL). The area below each Lorentzian curve is proportional to the concentration of particles of this particular subclass. The relative concentration of each subclass provides an estimation of the size distribution of HDL particles.

### Shen model

We used the classical Shen model of a spherical lipoprotein particle as a starting point to estimate the mean HDL sizes for each sample. The Shen model describes lipoproteins as spheres with a surface shell 2 nm thick consisting of phospholipids (PL), proteins (Prot) and free cholesterol (FC) covering a hydrophobic core of esterified cholesterol (EC) and triglycerides (TG). Once the concentrations of these molecules had been measured (see Table 1), their associated volumes could be determined. The ratio between the volume of the surface shell and the volume of the internal core is related to the mean lipoprotein size as follows:


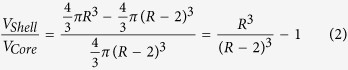


where the volume of the shell and the core was obtained from the concentration and the molecular volumes of each molecule:


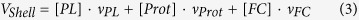



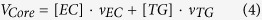


### Modified Shen model

The hypothesised discrepancies between the estimated mean sizes using the Shen model and the experimental ones, which were in agreement with the literature^19^,^23^,^24^, suggested that the lipid distribution within lipoprotein particles is not always well described by the Shen model. To solve these discrepancies, and on the basis that smaller particles have a higher V_shell_/V_Core_ ratio than larger particles, we modified the classical spherical model to obtain a ratio for smaller sizes so that some of the traditionally core lipids were located in the lipoprotein shell (increasing the V_shell_ and decreasing the V_Core_). The modified model is described in Equations 5, 6 and 7:


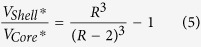



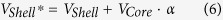



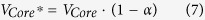


where *α* is the fraction of core lipids traditionally located in the surface shell.

Once the mean radii had been measured, *α* was determined for each sample as follows: first, we computed the geometric ratio between the volume of the external part (the 2 nm shell) and the internal part (the core) using the measured radius in nm; and then, we determined *α* by minimizing the difference between the biochemical ratio (right side of the Eq. 8) and the geometrical ratio (left side) so that some of the core lipids could be located in the surface shell.


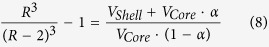


### Fluorescence study

Three different fluorescent probes – Prodan, Patman and Laurdan – were purchased from SIGMA-ALDRICH and used to evaluate the surface properties of lipoproteins. The fluorescent probes were introduced into the samples as previously reported^27^. The fluorescence spectra of the probes in native HDL lipoproteins were recorded on a luminescence spectrofluorometer AMINCO Bowman Series 2 with a Quarzglas cuvette (Hellma), and the excitation wavelength was 350 nm. The fluorescence spectra were normalized with the maxima intensity equal to one.

### AFM experiments

We used atomic force microscopy to measure the adhesion force between the tip of the AFM probe and the surface of the HDL particles on the basis of previously reported literature^35^,^36^. We obtained the topographic images as well as the adhesion force images by using the tapping force mode of AFM. The spring constant of the cantilever was 0.31 N/m, the peak force amplitude was 100 nm and the peak force frequency was 2 KHz. We observed three samples and about 15 HDL particles per sample were measured. In order to quantitate the particle size and relate it to the adhesion force, the images were initially processed with the NanoScope software, Ver. 1.20r1sr3 (Veeco), and then exported to MATLAB, Ver. 7.9.0.529 (The MathWorks).

### Statistical analysis

We performed two different statistical tests to detect differences between the variables studied: the mean size of the HDL fraction; the distribution between large, medium and small particles; and the distribution of the lipids traditionally found in the core (EC and TG) present in the lipoprotein shell. A statistical Mann-Whitney U test (two-tailed) was performed to identify significant differences between the CT and DM2 groups. This was followed by a Wilcoxon signed-rank test (two-tailed) to evaluate the treatment effects for paired samples (n = 24). In the fluorescence experiments, a statistical Mann-Whitney U test (two-tailed) was performed to identify significant differences between the maximum position of the CT and DM2 spectra and a principal component analysis (PCA) was carried out using the emission spectra of the fluorescent probes as input variables.

## Additional Information

**How to cite this article**: Amigó, N. *et al*. Lipoprotein hydrophobic core lipids are partially extruded to surface in smaller HDL: “Herniated” HDL, a common feature in diabetes. *Sci. Rep.* **6**, 19249; doi: 10.1038/srep19249 (2016).

## Supplementary Material

Supplementary Information

## Figures and Tables

**Table 1:** HDL biochemical composition (mg/dl) of CT population and DM2 patients.

	CT (26)	DM2 (29)	p
TG	11.1 [10.2, 12.9]	13.1 [11.1, 16.9]	0.012
EC	28.5 [21.3, 32.3]	17.8 [14.6, 22.1]	<0.001
FC	10.0 [8.5, 12.2]	8.2 [6.2, 9.5]	0.003
PL	59.7 [49.1, 68.9]	45.0 [41.6, 50.1]	0.002
Prot	113.0 [97.8, 127.8]	98.5 [87.0, 105.0]	0.007

Data are expressed as median ± 25–75. TG: triglycerides, EC: esterified cholesterol, FC: free cholesterol, PL: phospholipids, Prot: protein.

**Table 2:** HDL size and HDL lipid distribution between CT population and DM2 patients.

	CT (n = 26)	DM2 (n = 29)	p
Radius (nm)	4.7 [4.6, 4.8]	4.5 [4.4, 4.6]	0.002
% Core lipids in the surface	12 [0, 20]	20 [13, 27]	0.016
Relative Volume (%)			
Surface volume			
Core lipids	3 [0, 6]	6 [4, 7]	0.011
Prot	53 [51, 56]	54 [51, 56]	0.679
PL	37 [34, 39]	34 [31, 37]	0.022
FC	7 [6, 8]	6 [5, 7]	0.010
Internal core			
TG	30 [26, 37]	43 [36, 48]	<0.0001
EC	70 [63, 74]	57 [52, 64]	<0.0001

The table shows the mean HDL radius for the CT and the DM2 groups, the percentage (%) of the traditional core lipids (CL) located in the surface shell, and the percentage (%) of the surface volume occupied by the CL, the protein, the phospholipids and the free cholesterol; and the percentage (%) of the inner core volume occupied by the triglycerides and cholesterol. All the results are expressed as medians ± 25–75. Prot: protein, PL: phospholipids, FC: free cholesterol, TG: triglycerides, EC: esterified cholesterol.

**Table 3:** Treatment effects on the HDL biochemical composition (mg/dl) of DM2 patients.

	DM2	FF	p_1_	Ni	p_2_	p_3_
TG	13.3 [11.4, 17.4]	12.9 [10.0, 16.7]	0.149	13.3 [11.4, 16.2]	0.192	0.702
EC	17.5 [14.6, 22.1]	15.5 [13.7, 22.0]	0.903	18.7 [14.5, 25.3]	0.159	0.050
FC	8.5 [7.2, 9.5]	6.9 [5.6, 8.7]	0.027	7.9 [6.0, 10.5]	0.931	0.023
PL	48.1 [42.8, 51.9]	41.2 [37.6, 53.6]	0.079	40.4 [31.6, 56.0]	0.375	0.513
Prot	99.0 [86.8, 112.0]	103.0 [81.8, 114.5]	0.867	101.0 [87.8, 126.0]	0.651	0.366

Data are expressed as medians ± 25–75 of the 24 DM2 subjects who finished both interventions. Comparative between the basal state and the post fenofibrate state (p_1_) and the post niacin state (p_2_); comparative between treatments (p_3_). FF: fenofibrate, Ni: niacin, TG: triglycerides, EC: esterified cholesterol, FC: free cholesterol, PL: phospholipids, Prot: protein.

**Table 4:** Treatment effects on the HDL size and HDL lipid distribution of DM2 patients.

	DM2	FF	p_1_	Ni	p_2_	p_3_
Radius (nm)	4.5 [4.4, 4.6]	4.4 [4.4, 4.7]	0.305	4.6 [4.3, 4.7]	0.259	0.042
% Core lipids in the surface	20 [11, 28]	22 [16, 28]	0.578	15 [9, 25]	0.532	0.484
Relative (%) volume						
Surface volume						
Core lipids	5 [4, 7]	5 [4, 7]	0.958	4 [2, 8]	0.689	0.881
Prot	53 [50, 56]	55 [53, 59]	0.205	56 [53, 58]	0.170	0.958
PL	35 [33, 37]	33 [29, 37]	0.305	31 [29, 38]	0.170	0.434
FC	6 [5, 7]	5 [4, 6]	0.140	6 [6, 7]	0.434	0.159
Internal core						
TG	43 [37, 49]	40 [32, 48]	0.434	40 [31, 48]	0.205	0.217
EC	57 [51, 63]	60 [52, 68]	0.434	60 [52, 69]	0.205	0.217

The table shows the treatment effects on the mean HDL radius, the percentage (%) of the traditional core lipids (CL) located in the surface shell, and the percentage (%) of the surface volume occupied by the CL, the protein, the phospholipids and the free cholesterol; and the percentage (%) of the inner core volume occupied by the triglycerides and cholesterol. All the results are expressed as medians ± 25–75 of the 24 DM2 subjects who finished both interventions. Comparative between the basal state and the post fenofibrate state (p_1_) and the post niacin state (p_2_); comparative between treatments (p_3_). FF: fenofibrate, Ni: niacin, Prot: protein, PL: phospholipids, FC: free cholesterol, TG: triglycerides, EC: esterified cholesterol.
