# Supplementary material for: Lipoprotein hydrophobic core lipids are partially extruded to surface in smaller HDL: “Herniated” HDL, a common feature in diabetes
Source: Sci Rep. 2016 Jan 18;6:19249. doi: 10.1038/srep19249 (PMC4726105; doi:10.1038/srep19249)
Supplement: Supplementary Information [file srep19249-s1.pdf]

Supporting Information

Lipoprotein hydrophobic core lipids are partially extruded to surface in smaller HDL: "Herniated" HDL, a common feature in diabetes

Núria Amigó[1], Roger Mallol[1], Mercedes Heras[2], Sergio Martínez-Hervás[3], Francisco Blanco-Vaca[4], Joan Carles Escolà-Gil[4], Núria Plana[2], Óscar Yanes[1], *Lluís Masana[2], Xavier Correig[1]

1. Metabolomics Platform, Department of Electronic Electric and Automatic Engineering Universitat Rovira i Virgili, IISPV, Av. Universitat 1, 43204 Reus, Spain. Centro de Investigación Biomédica en Red de Diabetes y Enfermedades Metabólicas Asociadas (CIBERDEM), Madrid, Spain; 2. Vascular Medicine and Metabolism Unit, Research Unit on Lipids and Atherosclerosis, Sant Joan University Hospital, Universitat Rovira i Virgili, IISPV, C. Sant Joan s/n, 43201, Reus, Spain. CIBERDEM, Madrid, Spain; 3. Endocrinology and Nutrition Department, Hospital Clinico Universitario, CIBERDEM, INCLIVA, Department of Medicine, University of Valencia, C46010, Valencia, Spain; 4. Institut d'Investigacions Biomèdiques (IIB) Sant Pau, Antoni M. Claret 167, 08025 Barcelona, Spain. Departament de Bioquímica I Biologia Molecular, Universitat Autònoma de Barcelona, 08193 Bellaterra, Spain. CIBERDEM, Madrid, Spain.

S.1 From biochemical data to the analytical expression of the lipoprotein radius

The Shen model describes lipoproteins as spheres with a surface shell 2 nm thick consisting of phospholipids (PL), proteins (Prot) and free cholesterol (FC) covering a core of esterified cholesterol (EC) and triglycerides (TG). We used the Shen model and biochemical information to directly determine the theoretical radius of the lipoprotein particles as follows:

First, we computed the geometric ratio between the volume of the outside (the 2 nm shell) and the inside (the core). The geometric expression for a sphere of known radius is:

$$Ratio_{geometry} = \frac{\frac{4}{3}\pi \cdot R^3 - \frac{4}{3}\pi \cdot (R-2)^3}{\frac{4}{3}\pi \cdot (R-2)^3} = \frac{R^3}{(R-2)^3} - 1 \qquad (1)$$

Second, we found that this ratio was equal to the one obtained using the biochemical information and the Shen model:

$$Ratio_{bioq.} = \frac{V_{Exterior}}{V_{Interior}} = \frac{R^3}{(R-2)^3} - 1 \qquad (2)$$

where:

$$V_{Exterior} = V_{FreeCholesterol} + V_{Protein} + V_{Phospholipids} \qquad (3)$$

$$V_{Interior} = V_{EsterifiedCholesterol} + V_{Triglycerides} \qquad (4)$$

$$V_{molecule} = [molecule] \cdot v_{molecule} \qquad (5)$$

Finally, rearranging Eq. 2 we found the analytical expression for the radius:

$$R_s = \frac{20}{1 - \left(\frac{V_{Exterior}}{V_{Interior}} + 1\right)^{-\frac{1}{3}}} \qquad (6)$$

The molecular volumes were 1.058, 1.021, 1.102, 0.984 and 0.738 ml/mg for the EC, FC, TG, PL and Prot, respectively.

S.2 Correlation between triglycerides levels, size and percentage of core lipids in the surface.

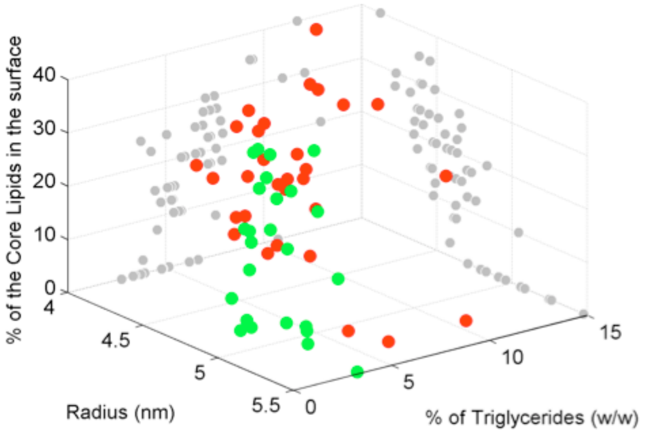

Figure S1 Percentage of the core lipids in the surface in relation to the size (nm) and the percentage of the triglycerides levels (w/w) of the HDL particles. The green circles represent the CT group and the red circles the DM2 group.

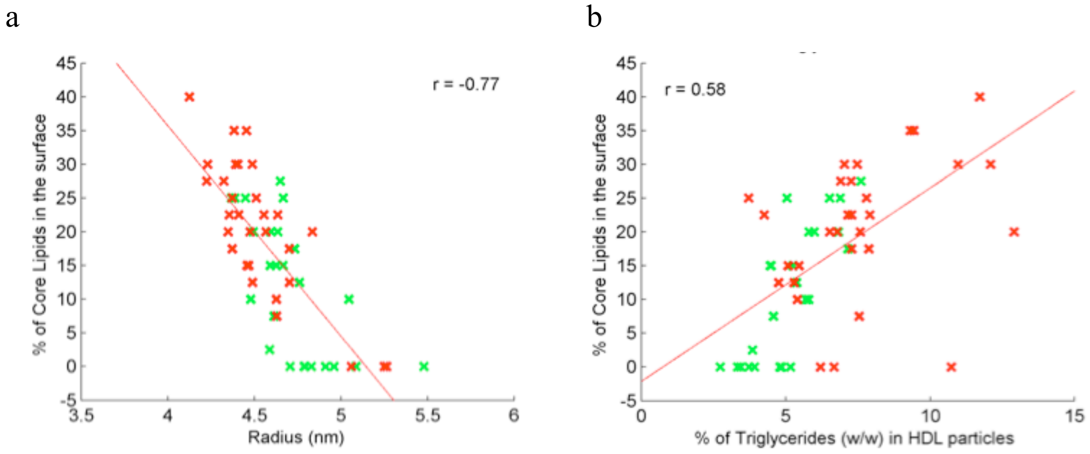

Figure S2. a) Percentage of the core lipids vs. Size. b) Percentage of the core lipids vs. TG. The green color represents the CT group and the red color, the DM2 group. R is the Pearson correlation coefficient.

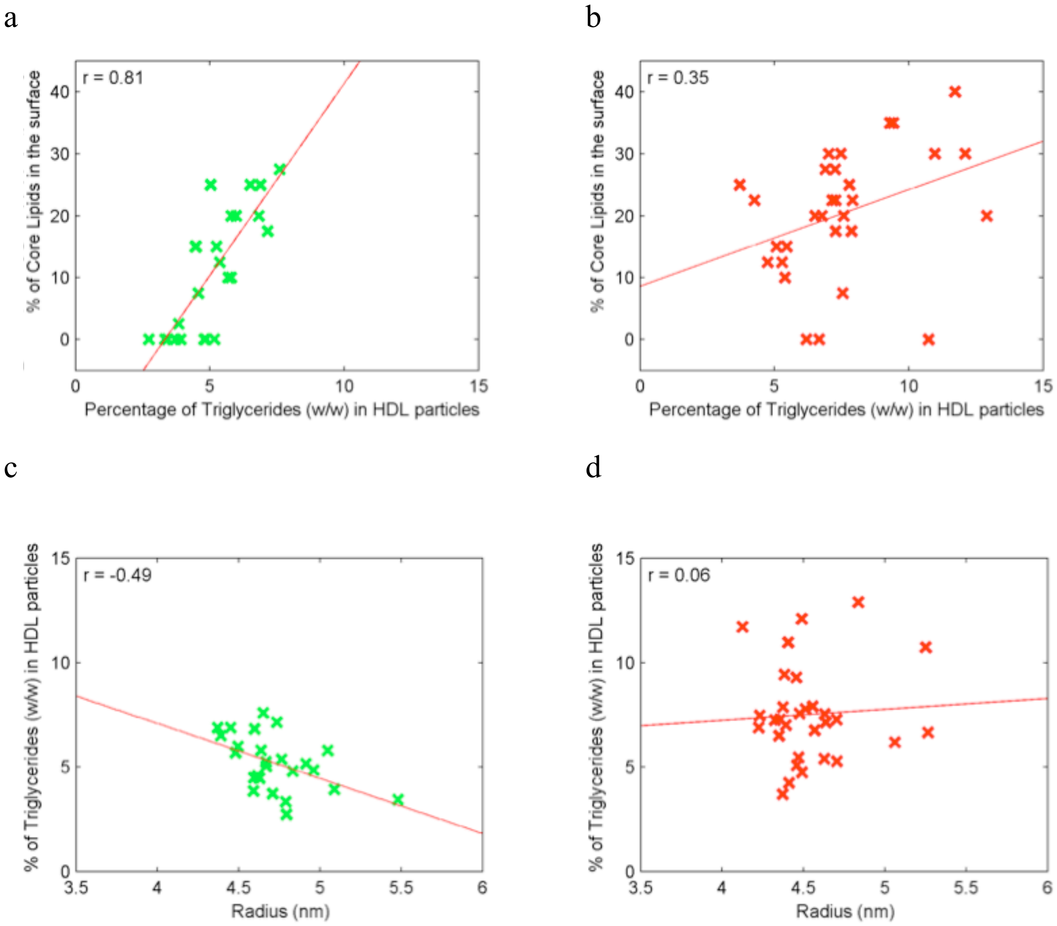

Figure S3. Scatter plots of the TG levels (w/w) and Percentage of the core lipids in the surface for a) CT group and b) for DM2 group; scatter plots of the Size (nm) and the TG levels (w/w) for c) CT group and d) for DM2 group. The green color represents the CT group and the red, the DM2 group. R is the Pearson correlation coefficient.

S.3 Fluorescence analysis

Fluorescence experiments were performed in a subgroup of the samples (CT n=8 and DM2 n=4) in order to observe differences in the surface polarity of the two groups. Three different fluorescent probes (Prodan, Patman and Laurdan) were used to evaluate the surface properties of lipoproteins.

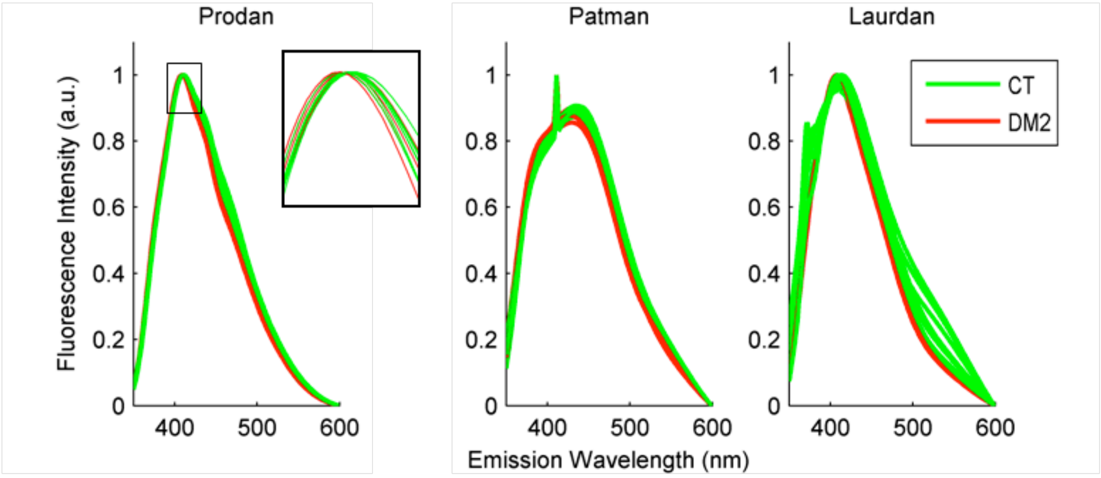

Figure S4. Fluorescence spectra of Prodan, Patman and Laurdan

The fluorescence spectra of all three probes showed the same tendency: the wavelengths of the emission maxima (λ_{em}) of the DM2 group were blue shifted (towards shorter wavelengths), indicating a more hydrophobic surface microenvironment than the CT group.

To evaluate the dispersion of the measurements and display the differences between the CT and the DM2 group, we performed a principal component analysis (PCA) of the fluorescence intensity between 350 and 600 nm of two repetitions per sample.

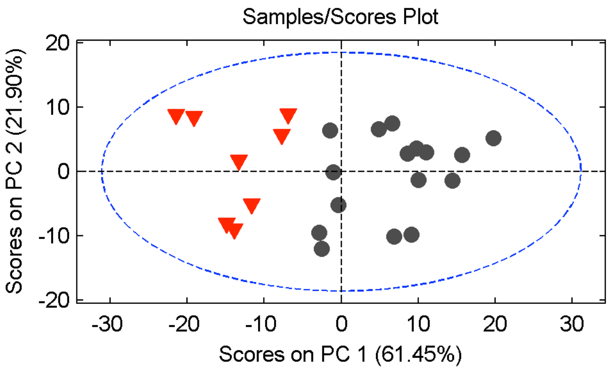

Figure S5. **Principal component analysis of the fluorescence raw data of two repetitions per sample.** The red triangles represent the DM2 group and the black circles, the CT group.

S.4 HDL subclass analysis

The biochemical composition of each subclass was calculated according to references [1-4], and the molecular weight of FC, CE, PL and TG was 387, 650, 787 and 885, respectively.

Table S1: Chemical composition of lipids and protein of each HDL subclass. Data are expressed as w/w. TG: triglycerides, CE: cholesteryl esters, FC: free cholesterol, PL: phospholipids, Prot: protein.

	Large HDL	Medium HDL	Small HDL
**FC**	7	4	3
**CE**	22	19	20
**PL**	30	30	20
**TG**	4	3	3
**Prot**	37	44	54

S.5 Atomic force microscopy experiments

We used atomic force microscopy to measure the adhesion force between the tip of the AFM probe and the surface of the HDL. We obtained topographic images and their adhesion force map. The adhesion force between the tip of the AFM probe and the sensed surface is inversely associated with the surface hydrophobicity.

Topographic image | Adhesion map

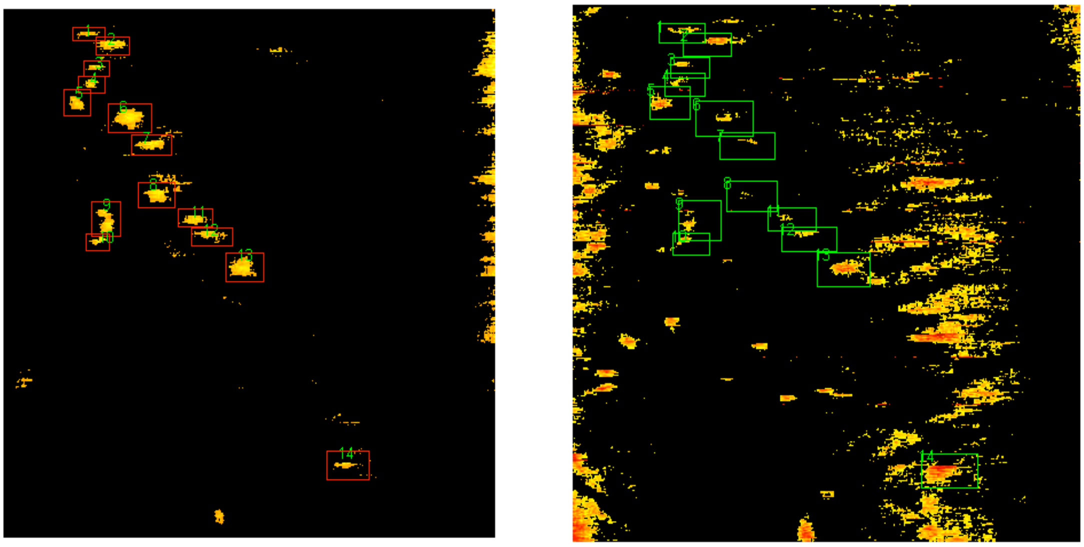

Figure S6. AFM topographic image and adhesion map of HDL particles. Lipoproteins of different size (red squares on the topographic image) interacted with the AFM tip. The adhesion map shows the adhesion force between the tip and the lipoprotein surface for each lipoprotein particle (green squares on the adhesion map).

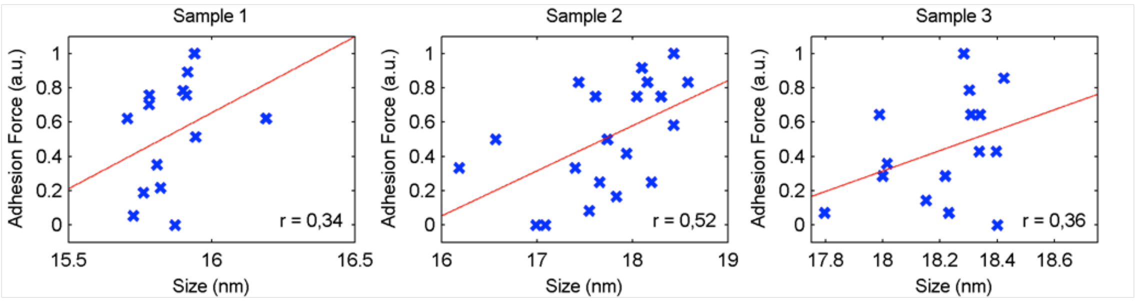

Figure S7. Scatter plots of HDL size by the tip adhesion force.

References

1. Kontush, A. & Chapman, M.J. Heterogeneity. in *High-Density Lipoproteins* 39-58 (John Wiley & Sons, Inc., 2011).
2. Nobecourt, E.*, et al*. Defective antioxidative activity of small dense HDL3 particles in type 2 diabetes: relationship to elevated oxidative stress and hyperglycaemia. *Diabetologia* **48**, 529-538 (2005).
3. Anderson, D.W., Nichols, A.V., Pan, S.S. & Lindgren, F.T. High density lipoprotein distribution. Resolution and determination of three major components in a normal population sample. *Atherosclerosis* **29**, 161-179 (1978).
4. Shuhei, N., Soderlund, S., Jauhiainen, M. & Taskinen, M.R. Effect of HDL composition and particle size on the resistance of HDL to the oxidation. *Lipids in health and disease* **9**, 104 (2010).
